# Autoantibodies against cytoskeletons and lysosomal trafficking discriminate sarcoidosis from healthy controls, tuberculosis and lung cancers

**DOI:** 10.1186/s43556-021-00064-x

**Published:** 2022-01-20

**Authors:** Samer Najeeb Hanoudi, Harvinder Talwar, Sorin Draghici, Lobelia Samavati

**Affiliations:** 1 Department of Computer Science, Wayne State University, Detroit, MI 48202 USA; 2 Department of Medicine, Division of Pulmonary, Critical Care and Sleep Medicine, Wayne State University School of Medicine, 3990 John R, 3 Hudson, Detroit, MI 48201 USA; 3 Center for Molecular Medicine and Genetics, Wayne State University School of Medicine, 540 E. Canfield, Detroit, MI 48201 USA

**Keywords:** T7phage library, Sarcoidosis, Tuberculosis, Microarray, Immunoscreening

## Abstract

Sarcoidosis is a systemic granulomatous disease of unknown etiology. Hypergammaglobulinemia and the presence of autoantibodies in sarcoidosis suggest active humoral immunity to unknown antigen(s). We developed a complex cDNA library derived from tissues of sarcoidosis patients. Using a high throughput method, we constructed a microarray platform from this cDNA library containing large numbers of sarcoidosis clones. After selective biopanning, 1070 sarcoidosis-specifc clones were arrayed and immunoscreend with 152 sera from patients with sarcoidosis and other pulmonary diseases. To identify the sarcoidosis classifiers two statistical approaches were conducted: First, we identified significant biomarkers between sarcoidosis and healthy controls, and second identified markers comparing sarcoidosis to all other groups. At the threshold of an False Discovery Rate (FDR) < 0.01, we identified 14 clones in the first approach and 12 clones in the second approach discriminating sarcoidosis from other groups. We used the classifiers to build a naïve Bayes model on the training-set and validated it on an independent test-set. The first approach yielded an AUC of 0.947 using 14 significant clones with a sensitivity of 0.93 and specificity of 0.88, whereas the AUC of the second option was 0.92 with a sensitivity of 0.96 and specificity of 0.83. These results suggest robust classifier performance. Furthermore, we characterized the informative phage clones by sequencing and homology searches. Large numbers of classifier-clones were peptides involved in cellular trafficking and cytoskeletons. These results show that sarcoidosis is associated with a specific pattern of immunoreactivity that can discriminate it from other diseases.

**Supplementary Information:**

The online version contains supplementary material available at 10.1186/s43556-021-00064-x.

## Introduction

Sarcoidosis is a granulomatous disease of unknown etiology [1], yet the unifying environmental or genetic factors as initiators of this disease have not been found [2–5]. Sarcoidosis affects multiple organs, such as the mediastinal lymph nodes, lungs, skin, CNS and the eyes [1, 2, 6, 7]. Other immunological features include a shift towards T helper type1 response, lymphopenia or neutropenia, and in some cases increased production of autoantibodies [8–11].

Sarcoidosis often coincides with other autoimmune disorders such as lupus erythematosus, vitiligo [9], autoimmune hepatitis, and Crohn’s disease (CD) [9, 12–14]. Several studies have suggested that the cellular and humoral responses associated with granuloma formation in this disease are the consequence of an exaggerated immune response to unknown antigens [15, 16]. Furthermore, subjects with sarcoidosis share several features, such as the presence of non-caseating granuloma, a lack of cutaneous reaction to tuberculin skin testing, and increased local and circulating inflammatory cytokines [1, 6, 7]. Interestingly, lack of responsiveness to PPD can also occur in other inflammatory diseases such as CD, rheumatoid arthritis (RA), or infectious diseases such as leprosy [5, 17, 18]. Pulmonary sarcoidosis and active pulmonary Tuberculosis (TB) share a number of clinical, radiological and histological similarities making differential diagnosis challenging.

Hypergammaglobulinemia, widely regarded as non-specific, is a frequent finding in sarcoidosis that may suggest active humoral immunity to unknown antigen(s) [10]. Targeted studies evaluating humural immunity in sarcoidosis have shown elevated IgG levels against components of various pathogens (*mycobacterium tuberculosis* and *propionibacterium acne*) [19, 20], as well as against several cellular commponents, including vimentin, a commponent intermediate filament protein, and others [21–23]. These data suggest the development of humoral responses against various antigens of different origins in this disease that can be profiled as diagnostics or to identify novel antigens contributing in pathogenesis of the disease.

The prevalence of sarcoidosis is higher in the northern hemisphere. Furthermore, it has been reported that the incidence of sarcoidosis is increasing in the developing world and China [24, 25]. Therefore, the development of highly accurate diagnostic classifiers for the diagnosis of sarcoidosis has significance worldwide. To identify the sarcoidosis-associated antigens, we constructed four different T7 phage display cDNA libraries, two of which originated from sarcoid bronchoalveolar lavage (BAL) cells and white blood cells (WBCs). Two other cDNA libraries were derived from cultured human embryonic fibroblasts and splenic monocytes. We combined all 4 libraries into a complex sarcoidosis library (CSL). This novel complex library is custom made for the discovery of biomarkers of respiratory disorders, in particular for sarcoidosis [23, 26–28]. Recently, we have shown that our microarray technology detects specific classifiers for various respiratory diseases [23, 26–28]. In our previous work, applying the same technology, we identified specific biomarkers for sarcoidosis and Tuberculosis as well as cystic fibrosis. Here, we tested the hypothesis that this technology is able to identify the specific classifiers for sarcoidosis in early stages within a large heterogeneous group of study subjects, including, heathy controls, Tuberculosis and lung cancer.

## Results

### A panel of 1070 clones exhibit limited ability to class separate sarcoidosis immune reactivity from other diseases

**Fig. 1:**
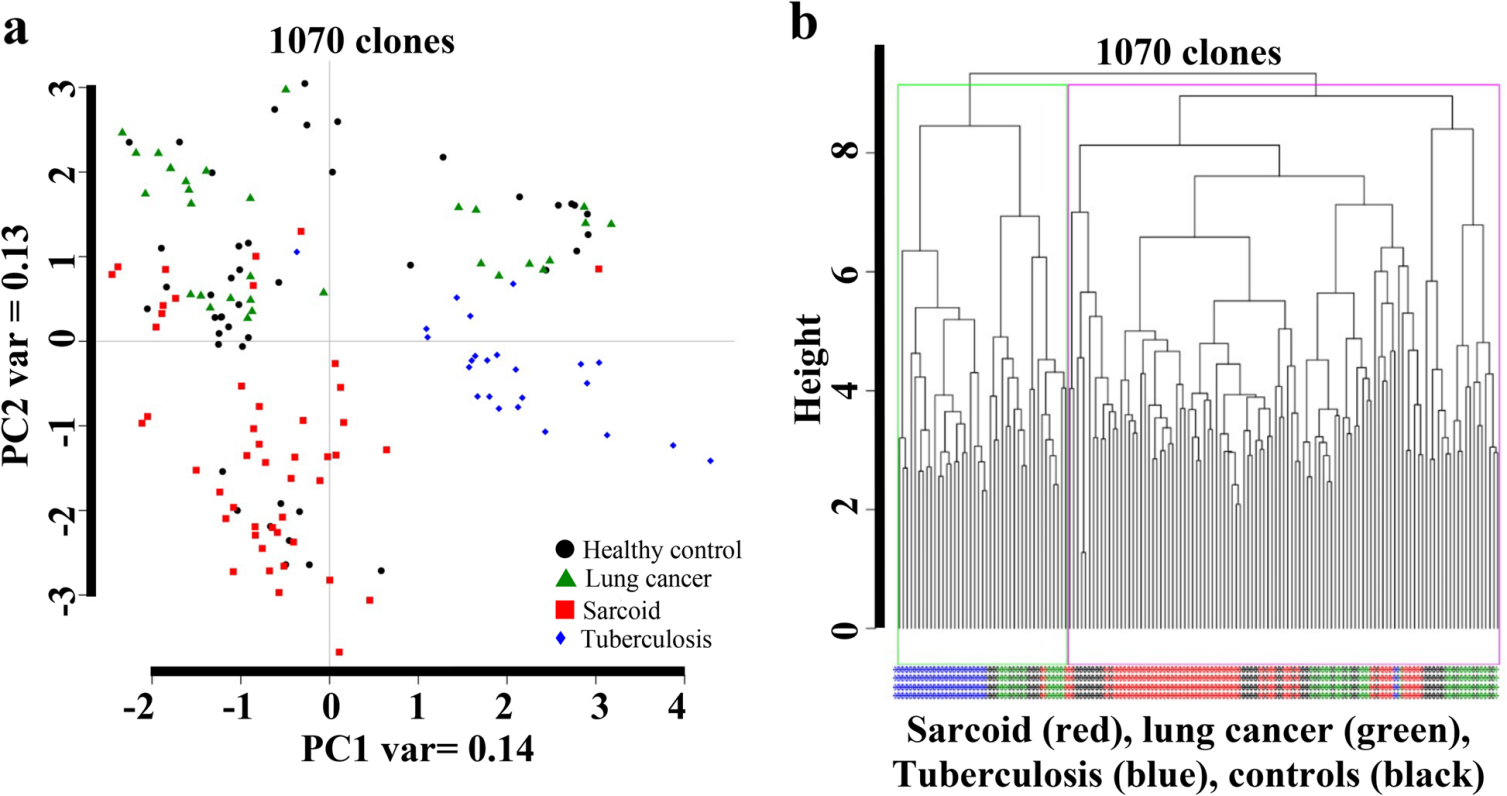
PCA and Hierarchal clustering (option1). **a** PCA plot along PC1 and PC2 generated with 1070 clones of four groups: (1) healthy control samples; (2) Sarcoidosis samples; (3) TB samples; and (4) Lung cancer. Biomarker clusters along the PC1 explain a variance of only 14%, while the variance along PC2 was about 13%. **b** The hierarchal clustering was applied on the healthy controls, sarcoidosis, TB patients and lung cancer with 1070 clones

From two highly enriched pools of T7 phage cDNA libraries through biopanning of the CSL library, we randomly selected 1070 potential antigens [23, 26, 27]. This antigen panel was used to construct microarray platform that was immunoscreened with 152 sera from diverse study subjects that included: healthy controls (*n* = 45); sarcoidosis (*n* = 52), smear-positive TB patients (*n* = 24), and lung adenocancer (LC) patients (*n* = 31). The demographics of the study subjects are shown in (Table 1). Following immunoreaction, the microarray data were pre-processed and then analyzed as previously described [23, 26, 27]. To assess the performance of 1070 clones, we performed an unsupervised principal component analysis (PCA) using all 1070 clones with data from 152 study subjects. As shown in Fig. 1a, several healthy controls and sarcoidosis patients were clustered with TB and lung cancer groups. We also performed unsupervised hierarchical clustering (HC) with all 1070 clones on these 152 samples. We observed the magenta cluster with a mix of samples and lacks specific sub-clusters of sarcoidosis samples (Fig. 1b). Figure 1a and b show that using all 1070 clones lacks the ability to class separate the sarcoidosis samples from other samples.

**Table 1:** Subjects demographics

Characteristic	Controls	Sarcoidosis	TB Subjects	Lung Cancers
**Age** (Mean ± SEM^a^)	40 ± 7.5	30.6 ± 11.8	40.5 ± 8.5	62.8 ± 11.8
**Gender**, N(%)	45	52	24	31
Male	12 (26)	11 (21)	14 (58)	13 (42)
Female	33 (74)	41 (79)	10 (42)	18 (58)
**Known ethnicity**, N(%)				
African American	31 (69)	49 (94)	0	0
African	0	0	4 (17)	0
White	0	3 (6)	0	31 (100)
Asians	14 (31)	0	20 (83)	0
**BMI** ^b^ (Mean ± SEM)	27 ± 3.8	28 ± 10.5	28 ± 6.9	28 ± 8.7
**Organ involvement**, N(%)				
Lung	NA^C^	48 (92)	24 (100)	31 (100)
Skin	NA	36 (69)	NA	NA
Neuro-ophthamologic	NA	31 (60)	NA	NA
Multiorgan	NA	45 (86)	NA	NA
**PPD** ^d^	NA	Negative	NA	NA
**TB smear** ^e^	NA	Negative	Positive	NA

^a^Standard error of mean, ^b^ Body mass index, ^c^ Not applicable, ^d^ Mantoux test (purified protein derivative), ^e^ TB Smear (AFB positive sputum)

### Improvement of class separation by decreasing the FDR threshold (option1)

To improve the classification performance, we applied two different t-tests: i) sarcoidosis training samples vs. healthy control training samples (option 1), and ii) sarcoidosis training samples vs. the rest (healthy controls, LC and TB samples (option 2). Two options resulted in two sets of differentially expressed clones. The first approach (option 1) at the threshold of False Discovery Rate (FDR) < 0.05 identifies 132 and at the threshold of FDR (< 0.01) identifies 14 significantly different clones between sarcoidosis and healthy controls.

**Fig. 2:**
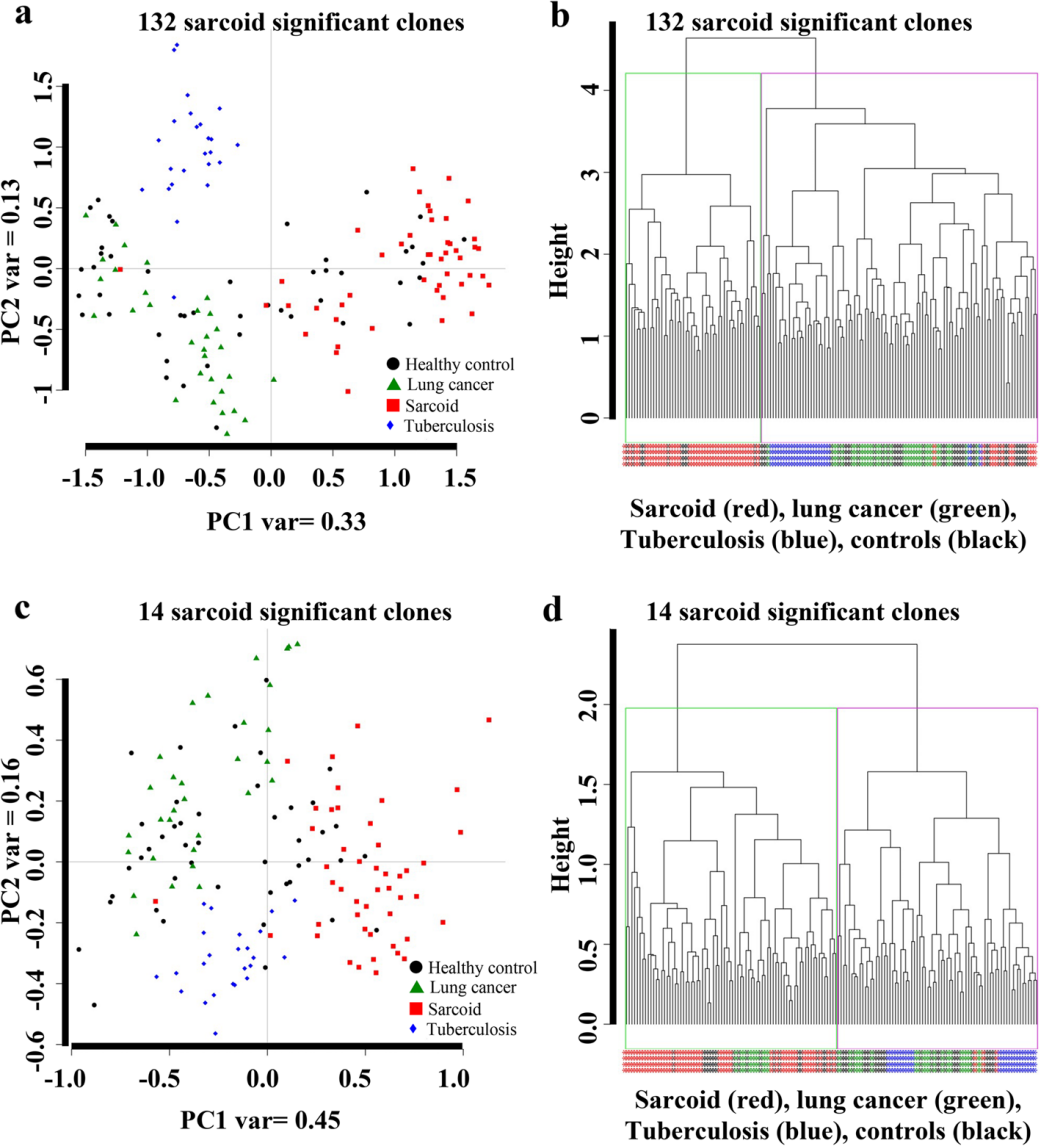
**a** PCA plot along the PC1 and PC2 results when applied on 132 sarcoidosis clones (option 1). The PC1 explained 0.33 of variance, whereas PC2 explained 13% of the variance. As shown, the sarcoidosis samples are well separated from the lung cancer, TB and most healthy control samples. **b** Hierarchal clustering using the top 132 sarcoidosis clones (FDR < 0.05). **c** PCA plot generated with the top 14 sarcoidosis classifier clones. The PC1 explained 45% of the variance, whereas PC2 explained 16% of the variance. **d** Hierarchal clustering using the top 14 sarcoidosis clones. This figure demonstrates better clustering performance with the 132 sarcoidosis clones and the top 14 classifier sarcoidosis clones (FDR < 0.01)

Two PCA plots were constructed to determine if the 132 significant clones (FDR < 0.05) and 14 clones (FDR < 0.01) from option1 can improve the class separation of sarcoidosis immune reactivity from healthy controls, TB and lung cancer samples. As shown in Fig. 2a, using 132 significant clones aided in an improved class separation of sarcoidosis subjects from all other groups with a variance of 33% along the PC1 (Fig. 2a). Similarly, using hierarchical clustering showed an improved separation of sarcoidosis samples from all the others (Fig. 2 b). Decreasing the FDR threshold to 0.01, we identified 14 highly significant clones differentially reactive in sarcoidosis versus healthy controls. When we constructed a PCA plot utilizing the 14 final clones from option 1, it resulted in a clear class separation of sarcoidosis samples from healthy controls, TB, and LC patients. The plot revealed a distinct discrimination along the PC1 direction that could explain 45 % of variance (Fig. 2c). Similarly, HC algorithm was applied using 14 clones, we observed a distinct hierarchical linkage separating sarcoidosis samples from other samples (Fig. 2d).

### Option 2 selected clones aid to a robust class separation

**Fig. 3:**
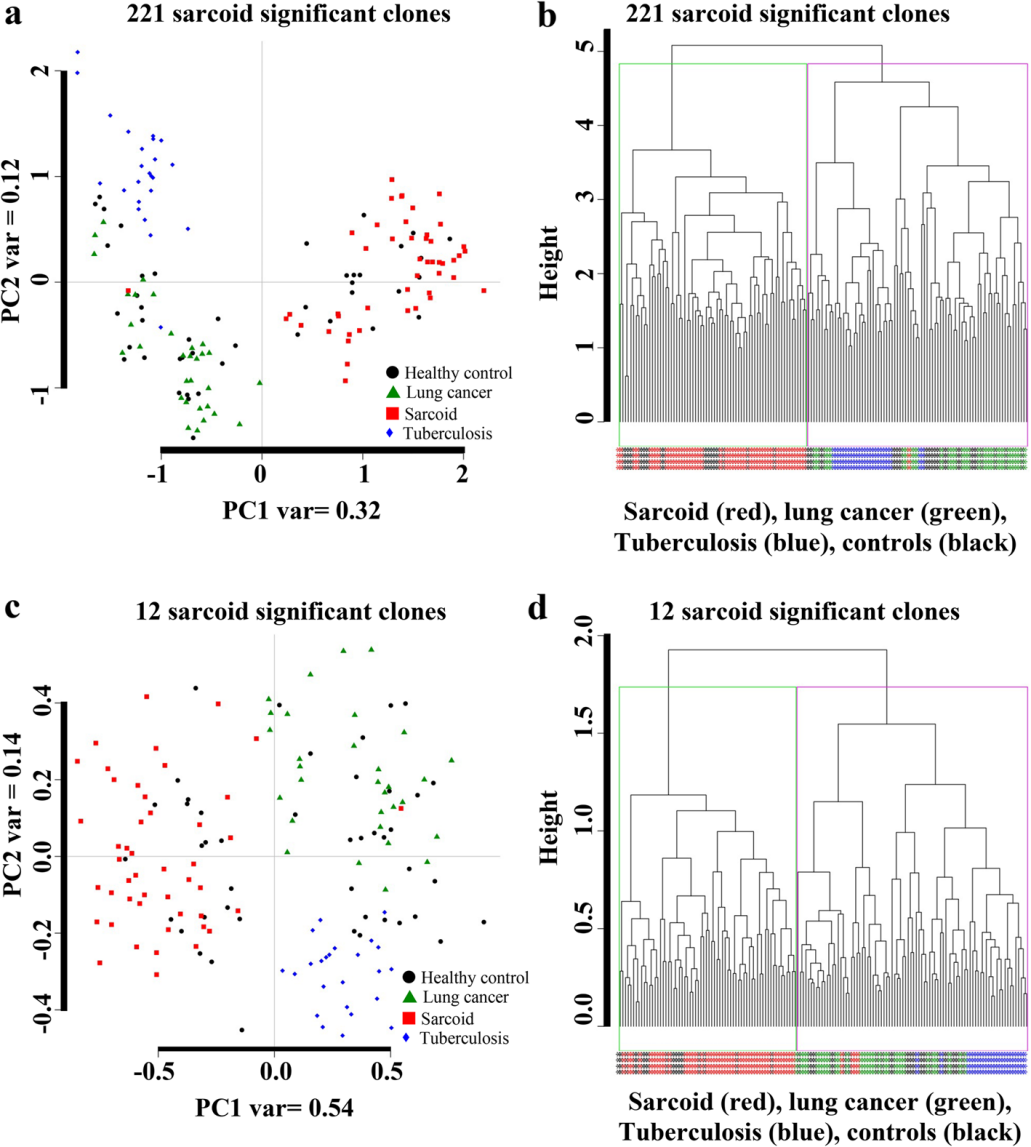
PCA and Hierarchal clustering (option 2). **a** PCA plot along PC1 and PC2 generated with 221 clones (FDR 0.01) of the four groups: (1) healthy control samples; (2) Sarcoidosis samples; (3) TB samples; and (4) Lung cancer. The PC1 explains a variance of 32%, while the variance along the PC2 was 12%. **b** The hierarchal clustering was applied on the healthy controls, sarcoidosis, TB patients and lung cancer with 221 clones (FDR < 0.01). **c** PCA plot along the PC1 and PC2 results when applied on the top 12 sarcoidosis classifier clones. The PC1 explained 54% of the variance, whereas PC2 explained 14% of the variance. As shown, the sarcoidosis samples are well separated from the lung cancer, TB and most healthy control samples. **d** Hierarchal clustering using the top 12 sarcoidosis clones. This figure demonstrates a robost class separation using top 12 sarcoidosis classifier clones

The statistical approach in option 2 yielded in 221 significant clones (FDR < 0.01) differentiating sarcoidosis from all other conditions. To demonstrate the performance of the clones identified through option 2 (sarcoidosis samples versus all other samples), we applied PCA and hierarchical clustering. As shown in Fig. 3a, using 221 clones aided in an improved class separation of sarcoidosis subjects from all other groups with a variance of 32% along the PC1. Similarly, when the hierarchical clustering algorithm was applied using 221 significant clones (FDR < 0.01), we observed a distinct hierarchical linkage nearly perfectly separating the sarcoidosis patients from TB and well separation from LC and healthy controls (Fig. 3b). Furthermore, we sorted the clones based on the *p*-values and chose top 12 reactive clones in option 2 to construct PCA plot and hierarchical clustering. As shown in Fig. 3 c and d, using the top 12 clones aided in a more robust class separation of sarcoidosis subjects from all other groups with a variance of 54% along the PC1 (Fig. 3c). A distinct hierarchical linkage is well separating the sarcoidosis samples from all other samples. The clustering analysis using the top 14 clones using option 1, and the top 12 using option 2 show a robust clustering of sarcoidosis samples from the rest (healthy controls, TB and LC).

**Fig. 4:**
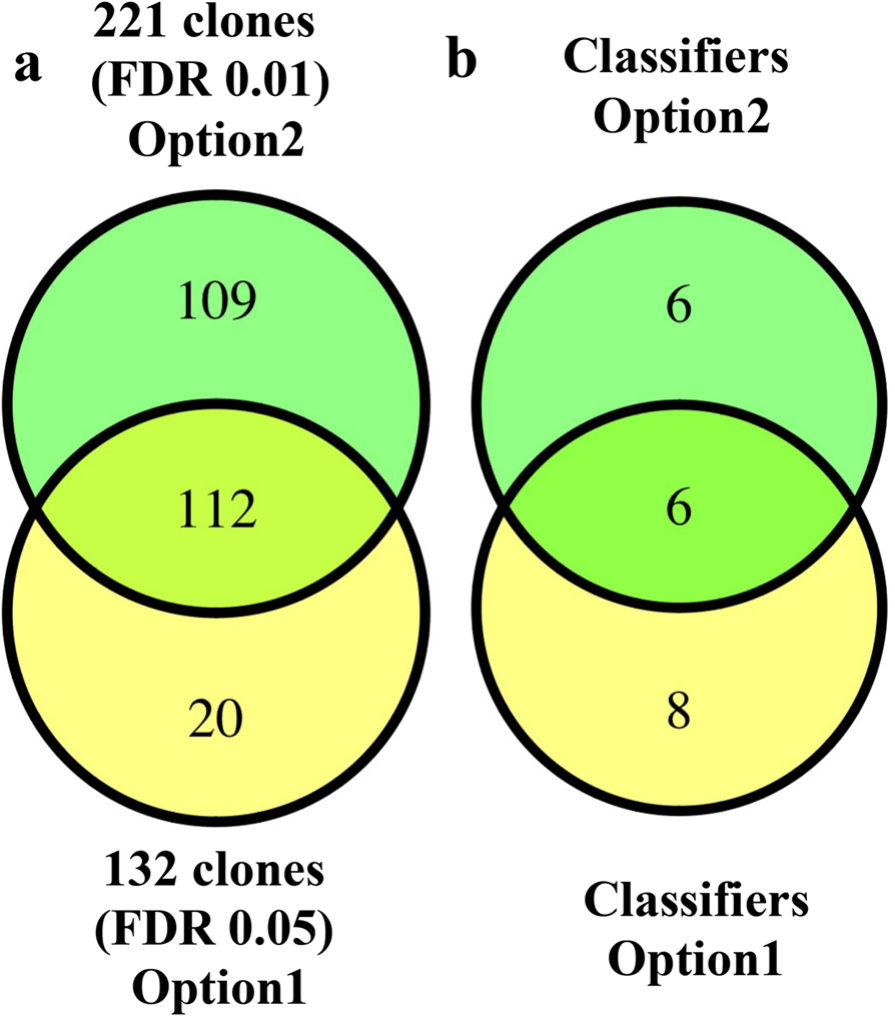
Diagrammatic representation of significant clones from two approaches (option 1 and 2). **a** Illustrates the Venn diagram of 132 clones (FDR < 0.05) from option 1 and 221 clones (FDR < 0.01) from option 2 and their intersection. **b** depicts the Venn diagram of the 14 classifiers clones from option 1 and 12 clones from option 2

Venn diagram illustrates the significant clones yielded through two different statistical approaches as well as their intersection (Fig. 4a and b). Figure 4a shows the Venn diagram of 132 clones (FDR < 0.05) from option1 and 221 clones (FDR < 0.01) from option 2. There were 112 shared clones between both options. Figure 4b shows the Venn diagram of the classifier clones identified in two statistical approaches (option 1 and 2 both at the FDR < 0.01). As shown, among 14 classifiers in option1 and 12 classifiers in option 2, six clones were common.

**Fig. 5:**
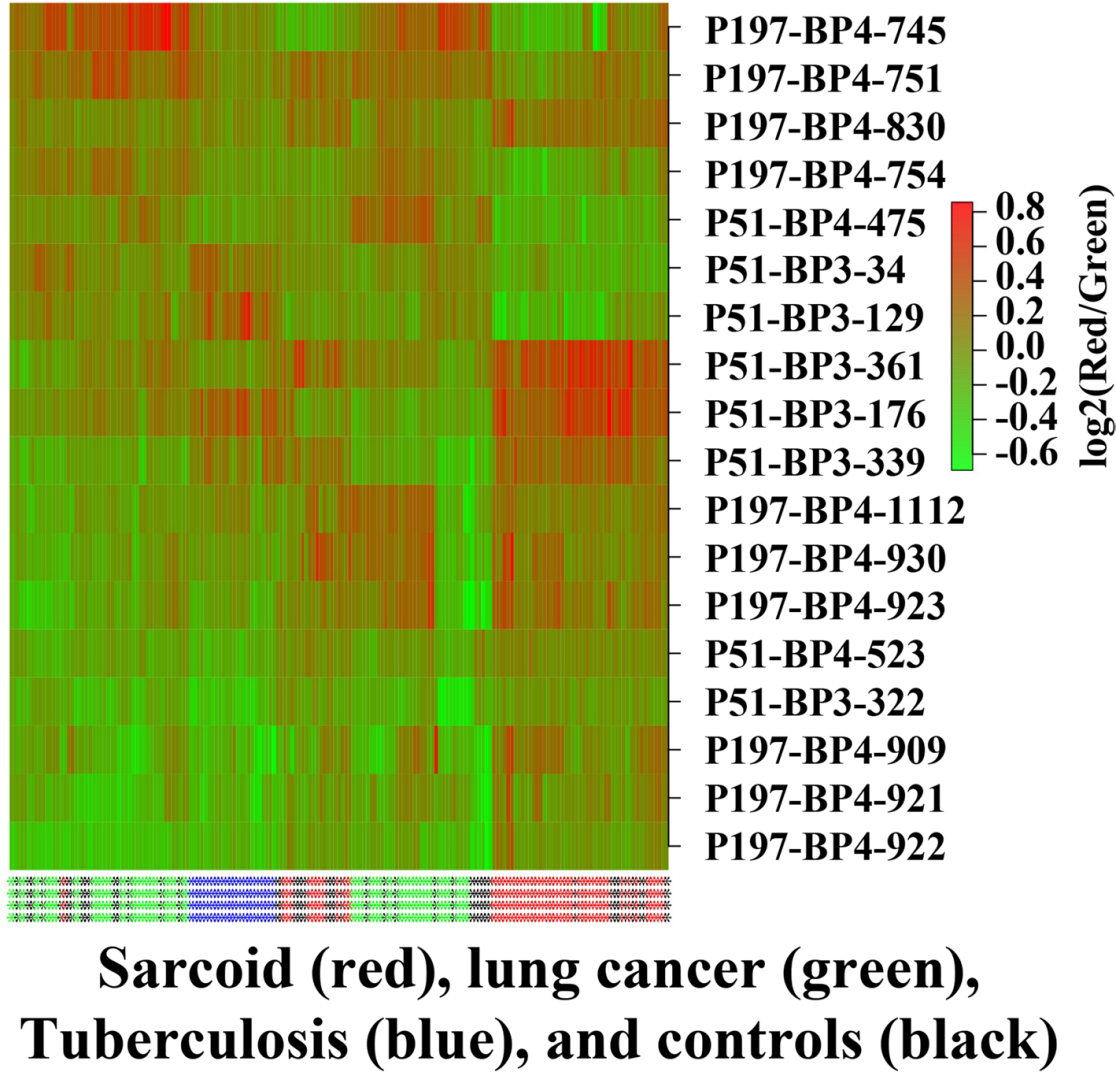
Displays a heatmap plot of the distinct expression features of the final clones identified in option 1 and 2

A heatmap plot (Fig. 5) displays the distinct expression features of the final classifier clones identified in options 1 and 2. The heatmap shows the profile for the classifier clones in all samples.

### Identification of classifiers to predict sarcoidosis

**Fig. 6:**
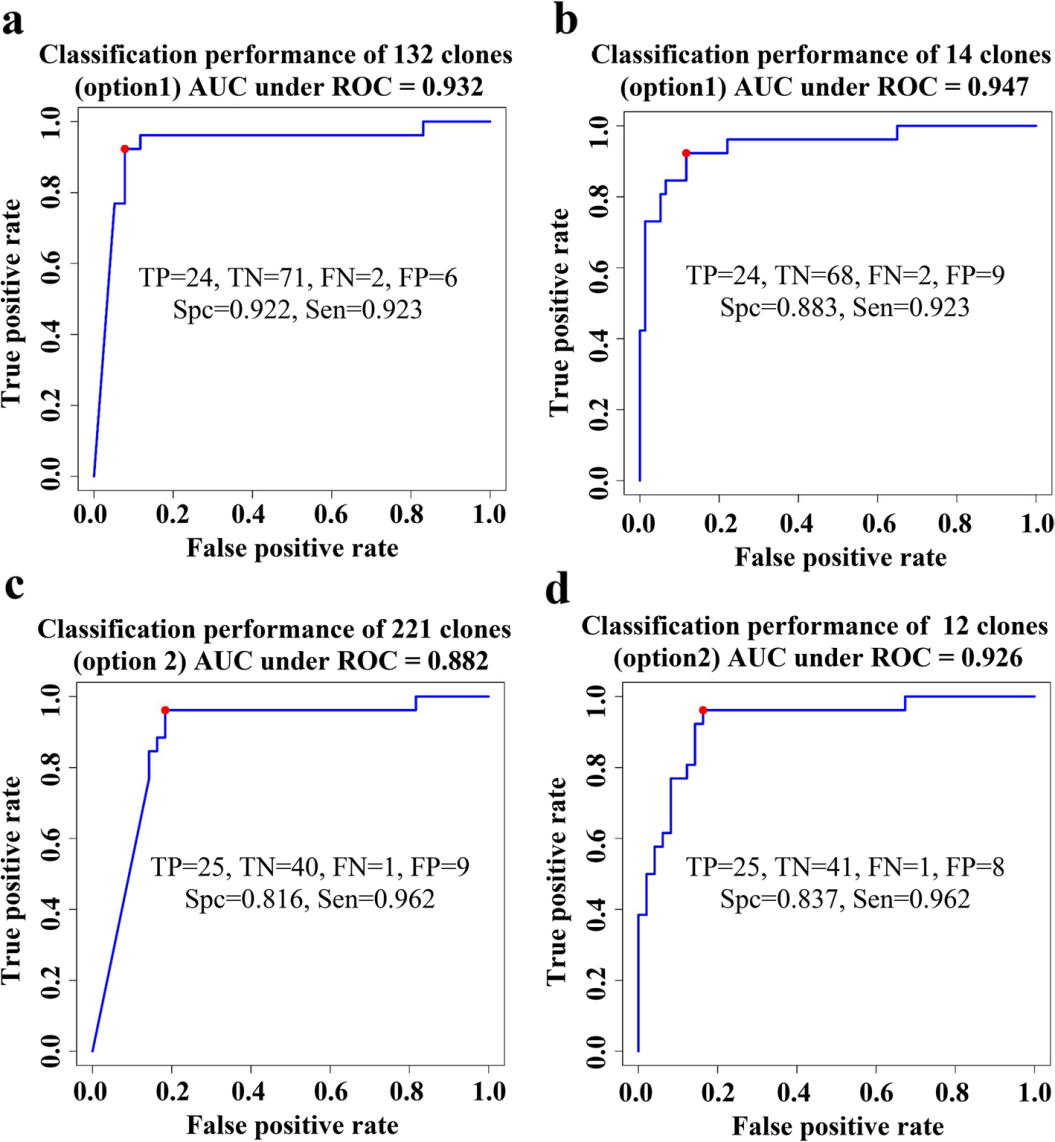
Classification to predict sarcoidosis from healthy controls, TB patients and LC patients on the testing set. **a** Performance of 132 clones on the testing set (option1). **b** Performance of the top 14 classifier clones on the test set (option1). The ROC curves demonstrate excellent classification performance with AUC of 0.947 with a sensitivity of 0.883 and specificity of 0.923. **c** Performance of 221 clones at the FDR = 0.01 (option2) on the testing set. **d** Performance of the top 12 classifier clones (option 2) on the test set. The ROC curves demonstrate strong classification performance with AUC of 0.926 with sensitivity of 0.962 and specificity of 0.837

To determine the classification performance of the identified clones using option 1 and 2, we applied the naïve Bayes classification method using option 1 and option 2 significant clones. We also assessed the classification performance of the top 14 clones from option1 and the top 12 clones from option 2. The classification models were trained on the training set and tested to classify sarcoidosis samples from other (healthy control, TB, and LC) on the testing set. As shown in Fig. 6a, the area under the curve (AUC) as a summary of the receiver operating curve (ROC) using the significant 132 clones (option1) was 0.932 with a true positive (TP) of 24, a true negative (TN) of 71, false negative (FN) of 2 and false positive (FP) of 6. Next, we applied the classifier model on the test set using the top 14 clones from option 1 (FDR < 0.01). The results of this analysis depicted in Fig. 6b, which shows an improved AUC of 0.947, when compared with the classification model of the 132 significant clones. Figure 6c, shows the classification results of the 221 significant clones (option 2) representing an AUC under the ROC of 0.882 with TP of 25, TN of 40, FN of 1 and FP of 9. Similar to option 1, we applied the classification model on the test set using the top 12 clones from option 2. The results of this analysis depicted in Fig. 6d, which shows an improved AUC of 0.926 when compared with the classification model of the 221 significant clones. These results suggest a robust classifier performance utilizing either the top 14 clones from option 1 or the top 12 clones from option 2.

### Characterization of sarcoidosis classifiers

Based on the results of training and test sets, we characterized the sarcoidosis classifier clones through sequencing. We sequenced classifiers’ clones and applied Expasy program to translate the cDNA sequences to peptide/protein sequences. Protein blast using algorithms of the BLAST program was applied to identify the highest homology to identified peptides [23, 27]. Furthermore, we compared these results with corresponding nucleotide sequences using nucleotide BLAST and determined the predicted amino acids in frame with T7 phage 10B gene capsid proteins. The identified clones were blasted with human genomes and then selected those specific peptide sequences that have the highest homology of amino acids sequence. After sequencing, we identified that two different DNA inserts were repeated twice. The selected peptide sequences of the final classifiers clones with the highest homology is shown in Table 2 shows the sarcoidosis clones identified by both statistical approaches (option 1 and option 2), gene names, sensitivity, specificity, and FDR adjusted *p*-values.

**Table 2:** Significant sarcoidosis classifiers

Clone ID	Protein names	Gene Name	FDR ^a^ Corrected ***p*** value	AUC ^b^	Sensitivity	Specificity
**P197-BP4–922**^**1&2**^	Cofilin 1 (non-muscle), isoform CRA_a	CFL1	8.10E-09	0.90	0.89	0.82
**P197-BP4–921**^**1&2**^	Chain A, Human Metap1	4FLI|A	2.70E-08	0.82	0.81	0.71
**P197-BP4–923**^**1&2**^	Inositol 1,4,5-trisphosphate receptor type 3	ITPR3	4.96E-07	0.80	0.85	0.63
**P197-BP4–1112**^**1**^	C-C motif chemokine 22 precursor	CCL22	3.68E-03	0.71	0.85	0.62
**P197-BP4–909**^**1**^	Chain A, Desmoplakin	DSP	5.45E-03	0.82	0.81	0.75
**P197-BP4–930**^**1**^	Ras-related protein Rab36 isoform 1	RAB36	6.30E-03	0.76	0.89	0.64
**P51-BP3–176**^**1**^	Apoptosis related protein APR-4, partial	PAR4	8.83E-03	0.75	0.81	0.70
**P51-BP4–523**^**2**^	Response gene to complement 32, isoform CRA_b	RGC32	1.70E-08	0.86	0.89	0.76
**P51-BP3–322**^**2**^	Probable C_mannosyltransferase DPY19L2 isoform X17	DPY19L2	1.57E-07	0.75	0.89	0.59
**P51-BP3–339**^**2**^ **P197-BP4–753**^**1**^	Receptor tyrosine-protein kinase erbB-4 isoform X1	ERBB4	4.96E-07	0.78	0.92	0.65
**P51-BP3–361**^**2**^	Neurite extension and migration factor	NEXMIF	5.16E-06	0.84	0.81	0.84
**P197-BP4–830**^**2**^	Solution structure of the F-actin binding domain of Bcr-Abl/c-Abl	1ZZP	8.54E-06	0.90	0.85	0.86
**P51-BP3–129**^**1&2**^	Interleukin 17A	IL17A	2.74E-07	0.87	0.81	0.80
**P197-BP4–745**^**1&2**^	SH3 domain-containing YSC84-like protein 1 isoform 4	SH3YL1	4.01E-07	0.84	0.73	0.88
**P197-BP4–754**^**1&2**^	Ras-related protein Rab-12	RAB12	1.57E-07	0.73	0.54	0.90
**P197-BP4–751**^**1**^	Transformation-related protein 10	TRG10	3.82E-03	0.67	0.65	0.69
**P51-BP4–475**^**1**^ **P51-BP3–57**^**1**^	Beta-polymerase	POLB	6.30E-03	0.80	0.65	0.91
**P51-BP3–34**^**2**^	INADL protein	INADL	7.11E-07	0.83	0.89	0.65

Clone ID: superscription 1&2 refers to the clone identified through option 1 or option 2, Protein name, NCBI gene name, ^a^ False discovery rate, ^b^ Area under the curve

## Discussion

Patients with sarcoidosis exhibit various immunological features including, a shift towards T helper type 1 response [29], lymphopenia or neutropenia, hypergammaglobulinemia, and in some cases increased autoantibodies [8–11]. Several studies have suggested that the cellular and humoral responses associated with granuloma formation in this disease are the consequence of an exaggerated immune response to unknown antigens [15, 16]. Numerous studies found components (RNA, DNA) of pathogens including *propionibacterium acnes* and *Mycobacterium tuberculosis* in sarcoidosis tissues [15, 16, 19, 30–32]. Similary, it has been shown that sarcoidosis blood monocytes react to TB antigens including, ESAT6 and KatG with increased interferon gamma production [33]. Sarcoidosis often coincides with other autoimmune disorders such as lupus erythematosus, vitiligo [9], and autoimmune hepatitis [9, 12–14]. Hypergammaglobulinemia is a frequent finding in sarcoidosis that may suggest active humoral immunity to unknown antigen(s) [10].

Using the serological analysis of antigens by recombinant expression cloning (SEREX) as a basis for autoantibody discovery, we critically examined the relevant methods of biomarker discovery and developed an innovative immunoscreening to optimize the identification of specific autoantibodies [23, 34, 35]. To achieve this goal, a heterologous sarcoidosis antigen library derived from mRNA of numerous sarcoidosis subjects were displayed on the T7 phage system [23, 28]. Furthermore, we used antibody recognition and random plaque selection during biopanning of the libraries to minimize the confounding effects of nonspecific antibodies. Recent evidence indicates that panels of biomarkers can achieve significantly higher accuracy than individual biomarkers [34, 36–40].

Previously, we have shown that our complex antigen library detects autoantibodies as biomarkers in sera of sarcoidosis, cystic fibrosis and MTB patients with high sensitivity and specificity as compared to healthy subjects [23, 26, 27]. Our current data indicates that our technology detects sarcoidosis classifiers in early stages of this disease as compared to various other lung diseases. Important to note that current sarcoidosis group differs from our previous study group. Sera were collected during initial diagnosis of sarcoidosis and none of patients were treated with corticosteroids or other immunosuppressive medications. Additionally, sera from TB patients differed from our previous study [23], as previous TB group were treated with antituberculosis medication. Furthermore, we performed two different statistical approaches to our data: Option 1, first detected the significant biomarkers between healthy controls vs. sarcoidosis; whereas option 2 chose the sarcoidosis clones by comparing sarcoidosis samples vs. all other groups. In both options, we used independent training and testing sets. Interestingly, 6 antigen clones were identical between option 1 and 2. Option 1 yielded in 8 unique clones, whereas option 2 yielded in 6 specific clones. Two sequences were repeated twice in two different clone IDs (Table 2).

Among 18 classifier clones, one clone (Chain A, Human Metap1) was repeated in both approaches. Importantly, in our previous published work, this sequence was also identified as sarcoidosis specific clone [23]. Human methionine aminopeptidase type 1 (hMetap1) is an enzyme well conserved from prokaryotes to eukaryotes. This enzyme cleaves N-terminal methionine on the client proteins and is important in cell cycle progression and angiogenesis [41]. Another repeated clone has homology to SH3YL1. Little is known about the role of SH3YL1 in human diseases or its role in immunity. Recent emerging data indicate that SH3YL1 regulates nicotinamide adenine dinucleotide phosphate (NADPH) oxidase (Nox) isozymes, thereby it modulates reactive oxygen species [42]. SH3YL1 promotes Dock4-mediated RAC1 (rhoGTPase) activation and cell migration [43]. RAC1 plays a role in cytoskeleton organization and autophagy. Interestingly, RAC1 also modulates mammalian target of rapamycine (mTor) [44]. Several studies highlighted the potential role of RAC/mTor and autophagy pathways in sarcoidosis [45, 46]. Other reports suggest that SH3YL1 regulates endosomal sorting complex required for transport (ESCRT) that is involved in endosome-lysosomal trafficking [47]. Further experiments need to elucidate the role of SH3YL1 in sarcoidosis. We identified two clones related to endo-lysosomal trafficking: one is ras-related protein RAB12 and another is ras-related protein RAB36. Both these proteins belong to Rab GTPase family. Recent evidence indicates GTPase family is involved in the complex membrane trafficking from endosome and lysosome, as well as plays essential roles in signaling that controls cell proliferation and differentiation [48]. RAB12 modulates mTORC1 activity and autophagy through regulation of the membrane trafficking from recycling endosomes to lysosomes [49]. RAB36 is largely conserved in vertebrates, yet it is a less well charactherized member of GTPase family. It has been shown that it interacts with Rab binding protein and is involved in vesicle trafficking to the lysosome, late endosome and Golgi apparatus [50, 51]. Sarcoidosis has been associated with various unfolded proteins, such as amyloid, therefore, it is conceivable that dysregulation of protein transport to the endosomes/lysosomes and autophagosomes play a significant role in this disease.

A relatively large peptide sequence (43AA) was identified with sequence homology to transformation related protein 10. This gene encodes a member of the bone morphogenetic protein (BMP) receptor family of transmembrane serine/threonine kinases. The ligands of this receptor are members of the TGF-beta superfamily. BMPs are involved in endochondral bone formation and embryogenesis. These proteins transduce their signals through the formation of heteromeric complexes of two different types of serine/ threonine kinase receptors: type I receptors of about 50–55 kD and type II receptors of about 70–80 kD [52]. For instance, mutations in BMP2 have been associated with primary pulmonary hypertension [53]. Another clone antigen was the colony stimulating factor 1 (isoform CRA_b). CSF-1 signals through its receptor (CSF-1R) promotes the differentiation of myeloid progenitors into heterogeneous populations of monocytes, macrophages, dendritic cells, and bone-resorbing osteoclasts [54]. Previously, we have shown a prominent role of monocytes and macrophages in sarcoidosis [55, 56]. A relatively small sequence had a homology with *erbB-4* gene. This gene is a member of the tyrosine protein kinase family and the epidermal growth factor receptor subfamily and is one of the four members in the EGFR subfamily of receptor tyrosine kinases. Three important antigen clones were related to cytoskeleton. We identified an antigenic peptide with 23 AA, which has homology to Cofilin 1. Cofilin family promotes actin filament disassembly and has been shown to be involved in myofibroblast differentiation [57]. Interestingly, when we used protein blast for all species, including all microorganisms this sequence had high homology with flagellin. Further investigation is needed to elucidate the role of this peptide sequence in sarcoidosis including fibrotic changes associated with this disease. Another related clone to cytoskeleton was the Chain A, Desmoplakin (DSP). DSP is a key junctional protein necessary for the morphogenesis and integrity of epithelial and vascular tissues and function as a linker protein providing attachment for cytoskeletal elements such as intermediate filaments [58]. The third peptide (clone = P197-Bp4–830) was related to F-actin binding domain of Ber/Abl/cAbl [59]. Two relatively small peptides had homology to C-C motif chemokine 22 (CCL22) and IL-17 R. CCL22 is produced by tissue-resident macrophages and modulates Th1/Th2 responses [60]. It has been shown that CCL22 regulates FOXP3+ regulatory T cells (Treg) [61]. Dysregulated Treg is well described in sarcoidosis [62]. IL-17R is the receptor for IL-17 but also plays a role in limiting the signaling pathway via the internalization of its ligand, thereby, it controls IL-17 pathway signaling [63]. Recent genetic study showed IL-17RA mutation in association with familial sarcoidosis [64]. We identified an epitope with a relatively large sequence (39AA) with homology to response gene to complement 32 (RGC32). RGC32 is induced by p53 in response to DNA damage and expressed in various tissues and is involved in numerous physiological and pathological processes, including cell proliferation, differentiation, fibrosis, metabolic disease [65]. The corresponding gene is involved in aniogenesis and regulated through hypoxia response element [66]. A sequence with 17aa had homology to probable C-mannosyltransferase DPY19L2, which mediates the C-mannosylation of tryptophan residues on client proteins, including type I cytokine receptors [67]. Two different clones (p51-BP4–457 and p51-BP3–57) with reduced expression in sarcoidosis had the same sequences with homology to POLB. POLB acts as a DNA polymerase is one of key enzymes for DNA repair [68]. Previously, autoantibody against POLB has been described in lupus erythomatsus [69]. This was experimentaly confirmed by mutation of POLB in mice that spontanously developed lupus like syndrome [70]. Another clone had homology with INADL protein. INADL (genecard symbol *PATJ*) protein has multiple PDZ domains. PDZ domains interact as scaffold protein to organize multimeric protein complexes at the cell membrane and are involved in signaling, trafficking, and function of G protein-coupled receptors [71]. INADL protein is not well characherized in human diseases, yet it appears that this protein regulates the cytoskeleton dynamic and cellular tight junction [72]. Further experiments are needed to evaluate the role of this protein in granuloma formation or modulation of tight junction in sarcoidosis.

Because various drugs may affect the autoantibody production, in current study, we performed immunoscreening using a set of sera from sarcoidosis subjects with no prior treatment. In spite of this, we found several shared antigenic clones between non-treated subjects (current study) and our previous study, in which samples derived from subjects, who were treated with immunosuppressive medication. We found a sets of classifiers with different sensitivity and specificity, some show increased expression and others showed decreased expression. Because sarcoidosis is a chronic disease involving many organs, the variation of autoantibodies expression profile may differ in early stages versus later stages or in various organ involvement. Although natural antibodies may also be beneficial to remove and neutralize pathogens, autoantibodies can directly interact with FCγ receptors or Toll- like receptors to initiate or amplify inflammation and perpetuate autoantibody production. Pathogenic autoantibodies can protect or cause diseases via neutralization of self-antigens, opsonization, antibody-dependent cellular cytotoxicity, activation of the complement system, pro-inflammatory and anti-inflammatory effect. Because of their broad reactivity for a wide variety of microbial components, natural antibodies have a major role in the defense against infections. Because some IgG autoantibodies may function as neutralization of pathogenic processes, the identification of decreased autoantibodies may be useful as therapeutics. Severel studies, including our study indicate that FCγ receptors play a role in sarcoidosis [73]. The identification of autoantibodies in sarcoidosis is important, as they may contribute to the cause of disease. Furthermore, sarcoidosis specific immunoreactivity against identified antigen clones can be used to develop a direct ELISA test for detection of autoantibody in sarcoidosis as a diagnostic. Further works are needed to elucidate the role of identified antigen clones in clinical feature and organ involvement of this disease.

## Materials and methods

### Chemicals

All chemicals were purchased from Sigma-Aldrich (St. Louis, MO) unless specified otherwise. LeukoLOCK filters and RNAlater were purchased from Life Technologies (Grand Island, NY). The RNeasy Midi kit was obtained from Qiagen, (Valencia, CA). The T7 mouse monoclonal antibody was purchased from Novagen (San Diego, CA). Alexa Fluor 647 goat anti-human IgG and Alex Fluor goat anti-mouse IgG antibodies were purchased from Life Technologies (Grand Island, NY).

### Patient selection

This study was approved by the institutional review board at Wayne State University, and the Detroit Medical Center. Sera from four groups wereincluded in this study: 1) healthy volunteers; 2) sarcoidosis subjects, 3) patients with adenocarcinoma of the lungs; and 4) smear positive pulmonary TB patients. All study subjects signed a written informed consent. All methods were performed in accordance with the human investigation guidelines and regulations by the IRB (protocol No = 055208MP4E) at Wayne State University. Sera from patients with Tuberculosis were obtained from the Foundation for Innovative New Diagnostics (FIND, Geneva, Switzerland). All TB patients had smear positive sputum for *Mycobacterium Tuberculosis* .

### Serum collection

Blood samples were collected and stored at − 80°^C^ as previously described [23].

### Construction and biopanning of T7 phage display cDNA libraries

T7 phage display libraries from BALs, WBCs, EL-1 and MRC5 were combined to generate a complex sarcoid library (CSL) [23]. Differential biopanning to iliminate the non-specific IgG was performed using sera from healthy controls, and sarcoidosis sera were used for positive enrichment [23].

### Microarray construction and immunoscreening

Informative phage clones were randomly selected and amplified after four rounds of biopannings and lysates were arrayed in quintuplicates onto nitrocellulose FAST slides (Grace Biolabs, OR) using the ProSys 5510TL robot (Cartesian Technologies, CA). The nitrocellulose slides were hybridized with sera and processed as described previously [23].

### Sequencing of phage cDNA clones

Individual phage clones were PCR amplified using T7 phage forward primer 5′ GTTCTATCCGCAACGTTATGG 3′ and reverse primer 5′ GGAGGAAAGTCGTTTTTTGGGG 3′ and sequenced by Genwiz (South Plainfield, NJ), using T7 phage sequence primer TGCTAAGGACAACGTTATCGG.

### Data acquisition and pre-processing

After hybridization, the microarrays were scanned in an Axon Laboratories 4100 scanner (Palo Alto, CA) using 532 and 647 nm lasers to produce a red (Alexa Fluor 647) and green (Alexa Fluor 532) composite image. Cy5 (red dye) labeled anti-human antibody was used to detect human serum IgGs were reactive to peptide clones, and a Cy3 (green dye) labeled antibody was used to detect the phage capsid protein [23]. Using the ImaGene 6.0 (Biodiscovery) image analysis software, the binding intensity of peptides to IgGs in sera was expressed as *log*_*2*_ (red/green) fluorescent intensities. These data were pre-processed using the limma package in the R language environment [27, 74, 75] and the normexp method was applied to correct the background [27, 76]. The *LOESS* method was used for within array normalization [23, 76, 77]. The scale method was applied to normalize between arrays [76, 77]. The intensity ratio of a clone in active sarcoidosis divided by intensity ratio of the same clone from healthy control samples to determine the fold change.

### Statistical analyses

To detect differentially expressed antigens for sarcoidosis, we applied a two-tailed *t-*test correcting for multiple comparisons using the false discovery rate (FDR) algorithm with a threshold of either 0.05 or 0.01 FDR [1]. All significant clones were sorted in an increasing order. We approach two statistical analyses using two-tailed t-tests. In *Option 1*, we applied a *t-*test between sarcoidosis training samples versus healthy controls training samples. Out of the 52 sarcoidosis samples, 26 samples were randomly assigned to the training set and the other 26 samples to the testing set. The training and testing set for the 45 healthy controls were randomly assigned to 23 samples in training and 22 samples in test sets. In the testing set, we added 24 tuberculosis samples and 31 lung cancer samples.

In *option 2,* we randomly split the samples from all groups in half. We assigned the first half of 23 control, 26 sarcoidoses, 16 lung cancer, and 12 tuberculosis samples to the training set. We assigned the second half of 22 healthy controls, 26 sarcoidosis, 16 lung cancer, and 12 tuberculosis samples to the testing set. We applied a t-test between sarcoidosis training samples versus healthy controls, lung cancer, and tuberculosis training samples to identify significant clones. For both options, we assessed the performance of classifiers clones, by applying principal component analysis (PCA), agglomerative hierarchal clustering (HC), heatmap, and naïve Bayes classifier. The naïve Bayes classifier model was built on the training samples to predict sarcoidosis samples from others (healthy controls and tuberculosis and lung cancer) samples and tested the classification model on the testing set (samples not used in the training set).

## Supplementary Information

**Additional file 1.** Supplementary Table 1. Clone ranking, clone ID, sequence of peptides, NCBI protein names, region of peptide similarity and % sequence coverage.

## Data Availability

The data are available from the corresponding authors upon reasonable request.

## Abbreviations

AUC: Area under the curve
BAL: Bronchioalveolar Lavage
CD: Chron’s Disease
CSL: Complex sarcoidosis library
FDR: False Discovery Rate
HC: Hierarchal clustering
PCA: Principal component analysis
WBC: White Blood Cells
MTB: *Mycobacterium tuberculosis*
TB: Tuberculosis
ROC: Receiver operating curve
