# Supplementary material for: Autoantibodies against cytoskeletons and lysosomal trafficking discriminate sarcoidosis from healthy controls, tuberculosis and lung cancers
Source: Mol Biomed. 2022 Jan 20;3:3. doi: 10.1186/s43556-021-00064-x (PMC8770712; doi:10.1186/s43556-021-00064-x)
Supplement: Supplementary file 1 — Additional file 1. Supplementary Table 1. Clone ranking, clone ID, sequence of peptides, NCBI protein names, region of peptide similarity and % sequence coverage. [file 43556_2021_64_MOESM1_ESM.docx]

**Increased autoantibodies against cytoskeletons and lysosomal trafficking in sarcoidosis discriminate sarcoidosis from healthy controls, Tuberculosis and lung cancer**

Samer Najeeb Hanoudi^1$^, Harvinder Talwar ^2$^, Sorin Draghici ^1^ and Lobelia Samavati ^2,3^,*

^1^ Department of Computer Science, Wayne State University; Detroit; MI 48202, USA ei1875@wayne.edu (S.N.H.); sorin@wayne.edu (S.D.)

^2^Department of Medicine, Division of Pulmonary, Critical Care and Sleep Medicine, Wayne State University School of Medicine and Detroit Medical Center, Detroit, MI 48201, USA;

^3^ Center for Molecular Medicine and Genetics, Wayne State University School of Medicine, 540 E. Canfield, Detroit, MI 48201, USA

$ authors contributed equally to this work

Running Title: Classifiers in sarcoidosis

Address correspondence to: Lobelia Samavati, MD; Department of Medicine, Division of Pulmonary, Critical Care and Sleep Medicine, Wayne State University School of Medicine, 3990 John R, 3 Hudson, Detroit, MI 48201, USA. Tel.: 313-745-1718; Fax: 313-933-0562; E-mail: [lsamavat@med.wayne.edu](mailto:lsamavat@med.wayne.edu) or [ay6003@wayne.edu](mailto:ay6003@wayne.edu) Tel.: +313-745-1718; Fax: +313-933-

**Table describes, clone ranking, clone ID, sequence of peptides, NCBI protein names, region of peptide similarity and % sequence coverage.**

| **Rank** | **Clone**  **and**  **Peptide**  **size** | **Peptide**  **Sequences of mimotopes in-frame with T7 10B gene** | **Description of the sequences that mimotopes mimic** | **Region of similarity of peptide** |
| --- | --- | --- | --- | --- |
| **1** | **P197_BP4_922**  **(23aa**) | S A C L Q S L R T Q L L T W A L V G D V G Q P | **cofilin 1 (non-muscle), isoform CRA_a [Homo sapiens]**  Sequence ID: [EAW74448.1](https://www.ncbi.nlm.nih.gov/protein/EAW74448.1?report=genbank&log$=protalign&blast_rank=89&RID=VCW3BAVH015) | ID=7/7 (100%) Gaps=0/7 (0%) Length=149  Query 16 LVGDVGQ 22  LVGDVGQ  Sbjct 39 LVGDVGQ 45  LQSLRTQLLT |
| **2** | **P197_BP4_921**  **(16aa**) | AG I S R E L V D K L A A A L E | Chain A, Human Metap1  Sequence ID: [4FLI_A](https://www.ncbi.nlm.nih.gov/protein/4FLI_A?report=genbank&log$=prottop&blast_rank=4&RID=H3HUESMR013) | Id=11/11 (100%) Gaps=0/11 (0%)  Length=326 Query 6 ELVDKLAAALE 16 ELVDKLAAALE  Sbjct 310 ELVDKLAAALE 320 |
| **3** | **P197_BP4_923**  **(5aa)** | RKRRQ | **inositol 1,4,5-trisphosphate receptor type 3 [Homo sapiens]**  **Sequence** ID: [NP_002215.2](https://www.ncbi.nlm.nih.gov/protein/NP_002215.2?report=genbank&log$=protalign&blast_rank=16&RID=FMYC946G014) | Id=5/5(100%0 Gaps=0/5(0%) Length=267  Query 1 RKRRQ 5  RKRRQ Sbjct 2654 RKRRQ 2658 |
| **4** | **P197_BP4_1112**  **(8aa)** | S D S C P H R P | **C-C motif chemokine 22 precursor [Homo sapiens]**  **Sequence** ID: [NP_002981.2](https://www.ncbi.nlm.nih.gov/protein/NP_002981.2?report=genbank&log$=protalign&blast_rank=3&RID=FMX2VDCC014) | Id=7/8 Gaps=1/8 (12%) Length=93  Query 1 SDSCPHRP 8  SDSCP RP Sbjct 57 SDSCP-RP 63 |
| **5** | **P197_BP4_909**  **(21aa)** | S K N L Y S F Y T E A S I E L H L N S H S | **Chain A, Desmoplakin [Homo sapiens]**  **Sequence** ID: [3R6N_A](https://www.ncbi.nlm.nih.gov/protein/3R6N_A?report=genbank&log$=protalign&blast_rank=50&RID=FMWDD60N014) | Id=8/10(80%) Gaps=0/10(0%) Length=450  Query 11 ASIELHLNSH 20  AS+E H NSH Sbjct 35 ASVEQHINSH 44  chondroitin sulfate N-acetylgalactosaminyltransferase 1-like isoform X2 |
| **6** | **P197_BP4_930**  **(12aa)** | S S L G C C E C K S V R | **ras-related protein Rab-36 isoform 1 [Homo sapiens]**  Sequence ID: [NP_001336806.1](https://www.ncbi.nlm.nih.gov/protein/NP_001336806.1?report=genbank&log$=protalign&blast_rank=90&RID=FMWX599F015) | Id=6/6(100%) Gaps=0/6(0%) Length=357  Query 1 SSLGCC 6  SSLGCC Sbjct 352 SSLGCC 357 |
| **7** | **P51_BP3_176**  **(8aa)** | SEKHPHRP | **apoptosis related protein APR-4, partial [Homo sapiens]**  Sequence ID: [AAD31316.1](https://www.ncbi.nlm.nih.gov/protein/AAD31316.1?report=genbank&log$=protalign&blast_rank=34&RID=FMXYMMYB015) | Id=6/6(100%) Gaps=0/6(0%) Length=114  Query 2 EKHPHR 7  +KHPHR Sbjct 59 QKHPHR 64 |
| **8** | **P51-BP4-523 (39aa)** | TDSTPALLSATVTPQKAKLGDTKELEAFIADLDKTLASM | **Response gene to complement 32, isoform CRA_b [Homo sapiens]**  **Sequence**  **ID: EAX08664.1** | **Id=39/39(100%) Gaps=0/39(0%) Length=78**  **Query 1 TDSTPALLSATVTPQKAKLGDTKELEAFIADLDKTLASM 39**  **TDSTPALLSATVTPQKAKLGDTKELEAFIADLDKTLASM**  **Sbjct 40 TDSTPALLSATVTPQKAKLGDTKELEAFIADLDKTLASM 78** |
| **9** | **P51-BP3-322 (17aa)** | SSERNGQFPWPLKMFLT | **probable C-mannosyltransferase DPY19L2 isoform X17 [Homo sapiens]**  Sequence  ID:XP_011536520.1 | Id=6/6(100%) Gaps=0/6(0%) Length=421  Query 12 LKMFLT 17  LKMFLT  Sbjct 219 LKMFLT 224 |
| **10** | **P51_BP3_339**  **(7aa)** | K F F Q N L S | **receptor tyrosine-protein kinase erbB-4 isoform X1 [Homo sapiens]**  Sequence ID: [XP_016859066.1](https://www.ncbi.nlm.nih.gov/protein/XP_016859066.1?report=genbank&log$=protalign&blast_rank=1&RID=FJDKVCP2014) | Id=6/6(100%) Gaps=0/6(0%) Length=1349  Query 2 KFFQNL 7  KFFQNL Sbjct 1043 KFFQNL 1048 |
| **11** | **P51-BP3-361**  **(10aa)** | INTDSIKLIA | **neurite extension and migration factor [Homo sapiens]**  ID: NP_001008537.1 | Id=6/6 (100%) Gaps=0/6 (0%) Length=1516  Query 2 NTDSIK 7  NTDSIK  Sbjct 598 NTDSIK 603  830 |
| **12** | **P197-BP4-830**  **(9aa)** | SKNLYSFLY | **Solution structure of the F-actin binding domain of Bcr-Abl/c-Abl [Homo sapiens]**  Sequence ID: 1ZZP_A | Id=6/6(100%) Gaps=0/6(0%) Length=130  Query 2 KNLYSF 7  KNLYSF  Sbjct 59 KNLYSF 64 |
| **13** | **P51_BP3_129**  **(8aa)** | S V D C R T C C | **Interleukin 17A [Homo sapiens]**  Sequence ID: [AAH66253.1](https://www.ncbi.nlm.nih.gov/protein/AAH66253.1?report=genbank&log$=protalign&blast_rank=99&RID=VCTJPCE5014) | **Id=6/7(86%) Gaps=1/7 (14%) Length=155**  Query 1 SVDCRTC 7  SVDC TC  Sbjct 141 SVDC-TC 146 |
| **14** | **P197_BP4_745**  **(70aa**) | S N E A N R F S F I L V L R G C Y N F L F L W S L E G S C L I E R K E T N R K F Y D I R A Y D I L F G D T P R P A Q A E D L Y E I L D S L Y | **SH3 domain-containing YSC84-like protein 1 isoform 4 [Homo sapiens]**  **Sequence** ID: [NP_001269616.1](https://www.ncbi.nlm.nih.gov/protein/NP_001269616.1?report=genbank&log$=protalign&blast_rank=3&RID=FKANNS1101R) | Id=45/47(95%) Gaps=2/47(5%) Length=246  Query 24 SLEGSCLIERKETNRKFY--DIRAYDILFGDTPRPAQAEDLYEILDS 70  SLEGSCLIERKETNRKFY DIRAYDILFGDTPRPAQAEDLYEILDS Sbjct 67 SLEGSCLIERKETNRKFYCQDIRAYDILFGDTPRPAQAEDLYEILDS 113 |
| **15** | **P197_BP4_754**  **(25aa)** | D E I F T L K L I E G G A L G K C E V **M** R V E P S | **ras-related protein Rab-12 [Homo sapiens]**  **Sequence** ID: [NP_001020471.2](https://www.ncbi.nlm.nih.gov/protein/NP_001020471.2?report=genbank&log$=protalign&blast_rank=63&RID=FKAGBV18014) | Id=9/10(90%) Gaps=1/10(10%) Length=244  Query 1 DEIFTLKLIE 10  DEIF LKL++ Sbjct 194 DEIF-LKLVD 202 |
| **16** | **P197_BP4_753**  **(7aa)** | K F F Q N L S | **receptor tyrosine-protein kinase erbB-4 isoform X1 [Homo sapiens]**  Sequence ID: [XP_016859066.1](https://www.ncbi.nlm.nih.gov/protein/XP_016859066.1?report=genbank&log$=protalign&blast_rank=2&RID=WCJZ37KZ01N) | Id=6/6 (100%) Gaps=0/6 (0%) Length=1349  Query 1 KFFQNL 6  KFFQNL  Sbjct 1043 KFFQNL 1048 |
| **17** | **P197_BP4_751**  **(43aa)** | S V A V S Q D C T T A L H P G Q Q S E T L S Q K K K G L Q R X R Q D Y F F X L N L F F | **transformation-related protein 10 [Homo sapiens]**  Sequence ID: [AAQ18032.1](https://www.ncbi.nlm.nih.gov/protein/AAQ18032.1?report=genbank&log$=protalign&blast_rank=1&RID=VW8JW3NH013) | Id=18/24(75%) Gaps=0/24(0%) Length=56  Query 2 VAVSQDCTTALHPGQQSETLSQKK 25  VAVS+D AL PG QSET SQKK  Sbjct 27 VAVSRDRANALQPGLQSETPSQKK 50 |
| **18** | **P51_BP3_57**  **(18aa)** | G K Y N S T F T S S I I H N K N [M](https://web.expasy.org/cgi-bin/translate/dna_sequences?/work/expasy/tmp/http/seqdna.118426,3,81) K | **beta-polymerase [Homo sapiens]**  Sequence ID: [AAA60133.1](https://www.ncbi.nlm.nih.gov/protein/AAA60133.1?report=genbank&log$=protalign&blast_rank=35&RID=VW42AVWS013) | Id=8/11 (73%) Gaps=0/11(0%) Length=335  Query 7 FTSSIIHNKNM 17  FT S I NKNM  Sbjct 272 FTGSDIFNKNM 282 |
| **19** | **P51_BP4_475**  **(18aa)** | G K Y N S T F T S S I I H N K N [M](https://web.expasy.org/cgi-bin/translate/dna_sequences?/work/expasy/tmp/http/seqdna.118426,3,81) K | **beta-polymerase [Homo sapiens]**  Sequence ID: [AAA60133.1](https://www.ncbi.nlm.nih.gov/protein/AAA60133.1?report=genbank&log$=protalign&blast_rank=35&RID=VW42AVWS013) | Id=8/11 (73%) Gaps=0/11(0%) Length=335  Query 7 FTSSIIHNKNM 17  FT S I NKNM  Sbjct 272 FTGSDIFNKNM 282 |
| **20** | **P51-BP3-34**  **(25aa)** | SGSLEVRSCTPAWVTERNFISKKKG | **INADL protein [Homo sapiens]**  Sequence ID: [AAI42662.1](https://www.ncbi.nlm.nih.gov/protein/AAI42662.1?report=genbank&log$=protalign&blast_rank=8&RID=FGHRTY6G016) | Id=14/22 (64%) Gaps=3/22 (13%) Length=1181  Query 3 SLEVRSCTPAWVTERNFISKKK 24  SL S TPAWVTE + +SKKK  Sbjct 1158 SL---SSTPAWVTEQDSVSKKK 1176 |
